# Bose–Einstein Condensation of Polaritons at Room Temperature in a GaAs/AlGaAs Structure

**DOI:** 10.1021/acsphotonics.4c01992

**Published:** 2024-12-20

**Authors:** Hassan Alnatah, Shuang Liang, Qi Yao, Qiaochu Wan, Jonathan Beaumariage, Ken West, Kirk Baldwin, Loren N. Pfeiffer, David W. Snoke

**Affiliations:** † Department of Physics, University of Pittsburgh, 3941 O’Hara Street, Pittsburgh, Pennsylvania 15218, United States; ‡ Joint Quantum Institute, University of Maryland and National Institute of Standards and Technology, College Park, Maryland 20742, United States; § Department of Electrical Engineering, Princeton University, Princeton, New Jersey 08544, United States

**Keywords:** polaritons, microcavities, Bose−Einstein condensation, room-temperature condensation, thermal equilibrium

## Abstract

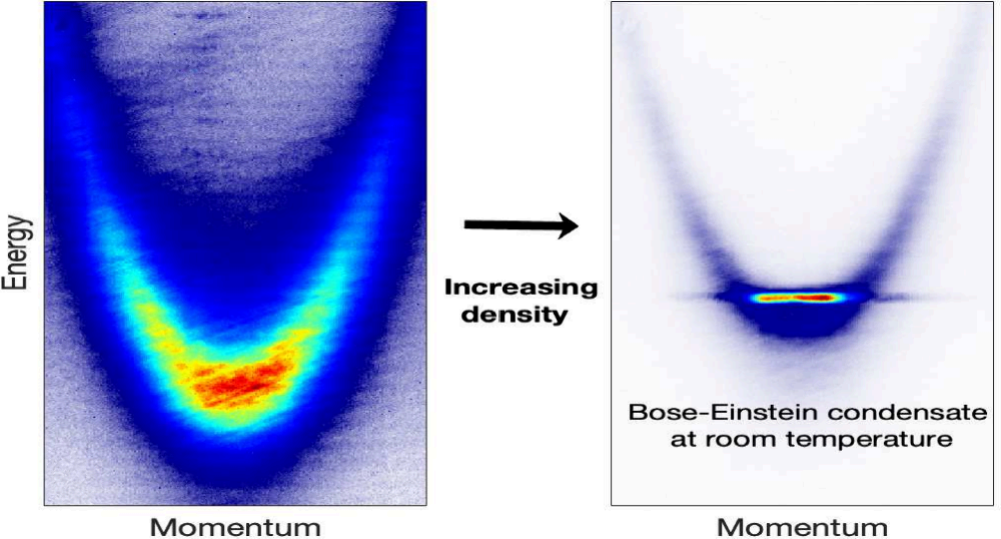

We report the canonical properties of the Bose–Einstein condensation of polaritons in the weak coupling regime, seen previously in many low-temperature experiments, at room temperature in a GaAs/AlGaAs structure. These effects include a nonlinear energy shift of the polaritons, showing that they are not noninteracting photons, and dramatic line narrowing due to coherence, giving coherent emission with a spectral width of 0.24 meV at room temperature with no external stabilization. This opens up the possibility of room temperature nonlinear optical devices based on polariton condensation.

## Introduction

While many clear demonstrations of effects of Bose–Einstein condensation of polaritons have been seen in GaAs-based structures at low temperature,^1−6^ it has generally been assumed that GaAs-based structures will not work for room-temperature condensates. This intuition is largely based on the binding energy of the excitons in GaAs, which is roughly 10 meV in quantum wells,^7^ well below the thermal energy scale at room temperature of *k*_B_*T* = 25.6 meV. However, it is not the case that polaritons can only be formed when excitons are fully stable bound states. Rather, the exciton-polariton is a quasiparticle state of the system that can coexist with free carriers and uncoupled excitons.

When there is light–matter coupling between excitons and photons, all that is needed for the existence of polaritons is an exciton resonance that lies reasonably near the energy of the photon mode. At room temperature, the exciton resonance in GaAs is broadened substantially due to phonon scattering, which tends to reduce the coupling between excitons and photons but does not completely destroy the coupling. A simple three-level model for our GaAs-based microcavity structures, which includes heavy-hole excitons, light-hole excitons, and photons, can be written as
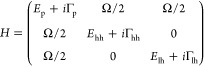
1where Γ_p_ gives the half-width at half-maximum of the photon line width broadening, here about ℏ/(100 ps) ∼ 0.01 meV, and Γ_hh_ and Γ_lh_ give the heavy-hole and light-hole exciton broadening, here set to 7.3 and 24.6 meV, respectively, based on measurements of the bare quantum well photoluminescence (PL) at room temperature (given in the Supporting Information). For a moderate exciton-photon coupling of Ω = 4.5 meV and our experimentally measured cavity photon and exciton energies, this gives a well-defined lower polariton line, with a half width of 0.69 meV and an exciton fraction of 8.5%. The degree of light–matter coupling of polaritons can be viewed as a continuum rather than just two extremes. In the extreme weak coupling regime (i.e., Ω ≪ Γ_ex_), the description of the eigenstates as polaritons is not useful. However, in the case of weak but nonzero coupling (i.e., Ω ∼ Γ_ex_), the bare photon states are not the proper eigenstates, and as seen in the above analysis, the eigenstates can possess a significant exciton fraction. In our case, the particles exhibit behavior more characteristic of polaritons than cavity photons, as evidenced by an observed blueshift. Therefore, we refer to them as weakly coupled polaritons, with, as we estimate, about 8.5% excitonic fraction.

In the experiments reported here, we used a GaAs/AlGaAs microcavity structure very similar to those of previous experiments,^8−10^ but in which the cavity photon energy falls very near the quantum well exciton energy at room temperature. We see all of the expected behavior for a polariton condensate, as seen previously in low-temperature experiments. This system has some aspects in common with pure photon condensates,^11,12^ but also important differences that arise from the interactions of the particles, in particular, strong nonlinearity. This opens up the possibility of optical transistor devices that operate at room temperature. The coherent emission also has an ultranarrow line width, without any active stabilization of the mode.

## Results and Discussion

The microcavity in this study consisted of a total of 12 GaAs quantum wells, with AlAs barriers embedded within a distributed Bragg reflector (DBR). The DBRs are made of alternating layers of AlAs and Al_0.2_Ga_0.8_As. The quantum wells are in groups of 4, with each group placed at one of the three antinodes of the 3λ/2 cavity. The large number of DBR periods gives the cavity a high *Q*-factor, resulting in a cavity lifetime of ∼135 ps and a polariton lifetime of ∼270 ps at resonance.^13^

With the sample at room temperature, we generated polaritons by pumping the sample nonresonantly with an M Squared wavelength-tunable laser, which was tuned to a reflectivity minimum, about 174 meV above the lower polariton resonance (740 nm). The laser focus was a Gaussian with full width at half-maximum (fwhm) ∼ 45 μm; to reduce heating of the sample, we modulated the pump laser using an optical chopper with a duty cycle of 1.7% and pulses of duration 41.6 μs, which is very long compared to the dynamics of the system. The nonresonant pump created electrons and holes, which scattered down in energy to become polaritons. The photoluminescence (PL) was collected using a microscope objective with a numerical aperture of 0.75 and was imaged onto the entrance slit of a spectrometer. The image was then sent through the spectrometer to a CCD camera for time-integrated imaging.

**Figure 1:**
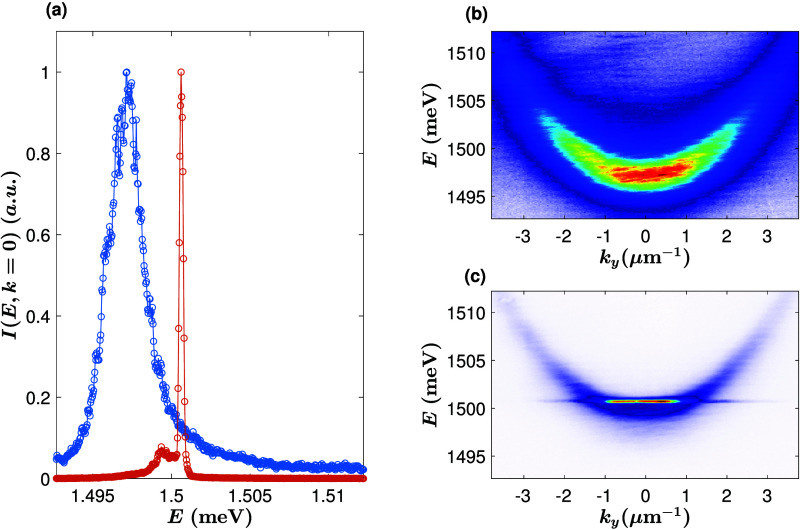
Ultralow line width. (a) The intensity at *k* = 0 at *P*/*P*_th_ = 0.8 (blue) and *P*/*P*_th_ = 1.34. The line width is extracted by fitting a Lorenzian, giving a line width of 2.5 meV for the blue curve and 0.24 meV for the red curve. (b) The polariton energy dispersion corresponding to blue curve in (a). (c) The polariton energy dispersion corresponding to red curve in (a).

Polariton condensation was observed as we increased the pump power across the BEC phase transition. The pump power needed was only about a factor of 2 higher than in the low temperature experiments in this type of structure, corresponding to a peak optical intensity of 1.5 × 10^4^ W/cm^2^, corresponding to an injected carrier density of approximately 5.7 × 10^22^ cm^–2^ s^–1^. As seen in Figure 1a, the line width becomes ultranarrow when the pump power is above the condensation threshold, reaching a line width of 0.24 meV (cf. previous work, which reported a line width of 0.7 meV^14^). Figure 1b,c shows that the spectral line narrowing is accompanied by momentum-space narrowing, as expected for a Bose condensate. The smaller peak in Figure 1a (red curve) arises from spatial inhomogeneity, with a laser pump spot that is most intense in the center. The polaritons first condense at the center of the spot where there is a strong blue shift due to repulsion from excitons generated by the pump, but some fraction of the polaritons flow away from the pump spot. The measured *E*(*k*) image represents an average measurement across real space, with the larger peak corresponding to the condensate at the top of the excitonic hill, while the smaller peak corresponds to the polaritons at the bottom of the potential-energy hill created by the excitons. This behavior has been seen in many prior experiments with polariton condensates.^15,16^

**Figure 2:**
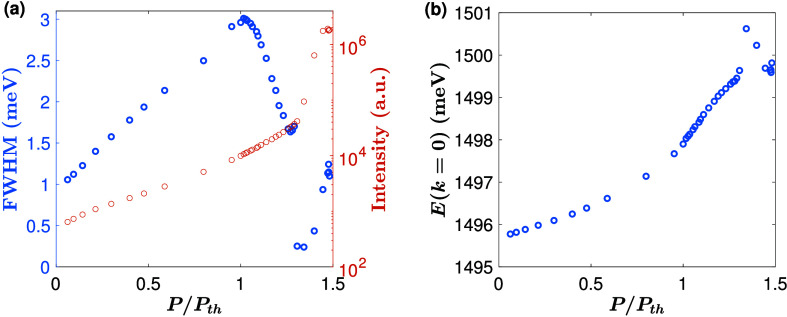
Blue shift and line width narrowing. (a) Full width at half-max at *k* = 0 and the intensity as a function of the pump power. (b) The blue shift at *k* = 0 as a function of the pump power.

A sharp nonlinear increase of the intensity by 2 orders of magnitude is also observed near the threshold of condensation. This nonlinear increase in intensity is accompanied by a factor of an ∼10 decrease in line width and significant energy blue shift (Figure 2), all of which are hallmarks of polariton Bose–Einstein condensation.^2,3^ The blue shift in particular shows that there is a nonlinear interaction of the polaritons with excitons, which are produced by the nonresonant pump. This confirms that the polaritons have a significant exciton component. Interestingly, while the system is in weak coupling, the nearness of the exciton line gives a substantial exciton fraction which gives a strong blue shift due to interactions. At the highest density, there is a slight red shift, which can be due to either lattice heating or depletion of the exciton cloud in the region of the condensate.^17^

**Figure 3:**
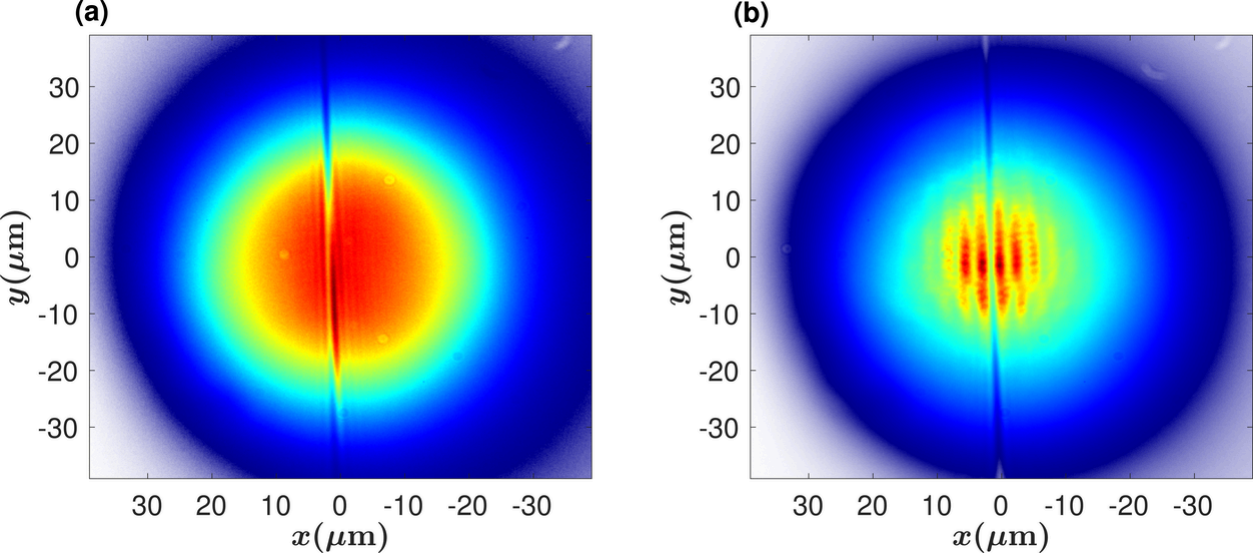
Coherence of polaritons. Real-space interference of the polaritons, recorded by superposing the spatial image of the emission with its mirror-image. (a) *P*/*P*_th_ = 0.17 and (b) *P*/*P*_th_ = 1.22.

In addition, we measured the coherence of the polariton by interfering with the electric field emitted from the cavity *E⃗*(*x*, *y*, *t*_0_) with its mirror image *E⃗*(−*x*, *y*, *t*_0_) using Michelson interferometry. We observed extended spatial coherence and an increase in the visibility as the pump power is increased (Figure 3).

We found that for photonic detunings, the polaritons tend to self-trap into the center of the pump spot for densities above the threshold of condensation, an effect which has been well observed at low temperature.^17^ This leads to multimode behavior of the condensate when it has mostly photonic character. To minimize this self-trapping effect, the data reported here were taken at a higher exciton fraction. The exciton fraction can be chosen by moving to different locations on the sample with different cavity photon energy.

**Figure 4:**
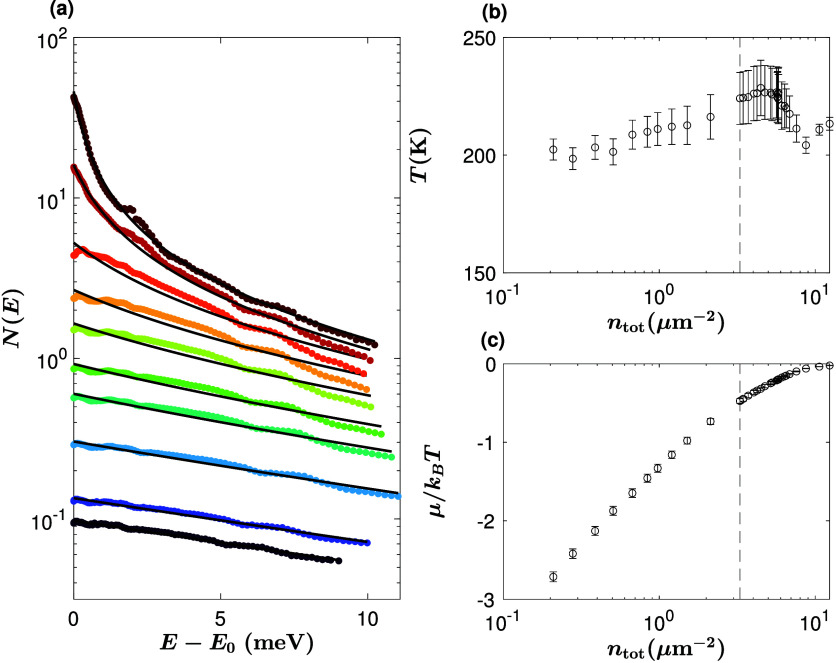
Thermalization of polaritons. (a) Occupation number of the polaritons as a function of energy. The solid lines are best fits to the equilibrium Bose–Einstein distribution. (b) the effective temperature and (b) the reduced chemical potential of the polaritons obtained from the fits to the Bose–Einstein distribution. The vertical dashed line in parts b and c denotes the critical density, defined as the total density of the polaritons at *P*/*P*_th_ = 1.

To study thermalization of the these polaritons, we used angle-resolved imaging to obtain the spectral function *I*(*k*, *E*), as done in previous works^8,10^ and described in the Supporting Information. This spectral function was then converted to the occupation number *N*(*E*) using a single efficiency factor. Figure 4 shows the distribution function of polaritons for different pump powers. We used a circular real space filter with a radius of ∼22 μm to collect light from a fairly homogeneous region of the PL. The measured occupation number was then fit to the Bose–Einstein distribution, given by
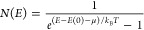
2where *T* and μ are fit parameters, which give the temperature and chemical potential of the polaritons, *E* is the polariton energy, and *E*(0) is the polariton ground state energy at *k* = 0, which shifts to higher energy as the density increases, due to many-body renormalization.^10^ We find that polaritons are well described by the Bose–Einstein distribution, as shown by the fits in Figure 4a. At low density, the Bose–Einstein distribution becomes a Maxwell–Boltzmann distribution, which is a straight line in the semilog scale. At high density, the Bose–Einstein statistics become important, giving rise to an upturn at ground-state energy *E*(*k* = 0).

Additionally, we plot the fitted values of temperature *T* (Figure 4b) and reduced chemical potential μ/*k*_B_*T* (Figure 4c) as a function of the total polariton density. Interestingly, the polaritons equilibrate to a temperature *T* ∼ 220 K lower than the reservoir *T* ∼ 293 K. This was also seen for noninteracting photons.^12^ Although it is surprising, we know that the polaritons are generally decoupled from the phonon and bath temperature; at low temperature, the polaritons are typically 20 K above the bath temperature. Cooling will occur whenever the most frequent downconversion of excitons into polaritons occurs into low-energy polariton states. A recent theoretical work^18^ predicted a simulated cooling effect, but as seen in Figure 4b, we observe effective polariton temperatures below room temperature even at very low pump density.

The critical density of condensation at room temperature can be estimated by relating the average distance between the particles to the de Broglie wavelength *a* ∼ λ_th_, which gives
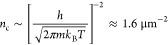
3where *m* = (3.96 ± 0.95) × 10^–5^*m*_*e*_ is the mass of the polaritons (measured from data like that of Figure 1b, and *T* ∼ 220 K is the temperature. This is within the uncertainty of the directly measured density using a calibrated light source to establish the absolute photon emission rate, which gives *n* = 2.97 ± 1.75 μm^–2^ for the polaritons at *P*/*P*_th_ = 1.

## Conclusions

Many material systems such as transition metal dichalcogenides,^19,20^ perovskites,^21,22^ and organics^23−25^ have been proposed and studied for room temperature Bose condensation for the purpose of nonlinear coherent device applications. Our observation of room temperature BEC here results shows that the same effects can occur in the well-studied system of GaAs and related III–V semiconductors, which can have extremely high quality and uniformity.

There are many commercial vertical-cavity surface-emitting lasers (VCSELs) with similar design to our structure, including the recent devices used for photon condensation.^12^ We believe that similar designs should be able to show the nonlinear behavior reported here, allowing for transistor-like operation^26^

## Supporting Information Available

Information about the polariton density calibration method, occupation extraction method, measurement of the exciton energy, and simulations of the three-level model, as well as additional data taken from a different location on the sample (PDF)
