# Supplementary material for: Bose–Einstein Condensation of Polaritons at Room Temperature in a GaAs/AlGaAs Structure
Source: ACS Photonics. 2024 Dec 20;12(1):48–52. doi: 10.1021/acsphotonics.4c01992 (PMC11741138; doi:10.1021/acsphotonics.4c01992)
Supplement: Supplementary file 1 — ph4c01992_si_001.pdf [file ph4c01992_si_001.pdf]

Supplementary Information: Bose-Einstein condensation of polaritons at room temperature in a GaAs/AlGaAs structure

Hassan Alnatah,*,† Shuang Liang,† Qi Yao,‡ Qiaochu Wan,† Jonathan Beaumariage,† Ken West,¶ Kirk Baldwin,¶ Loren N. Pfeiffer,¶ and David W. Snoke†

†*Department of Physics, University of Pittsburgh, 3941 O'Hara Street, Pittsburgh, Pennsylvania 15218, USA*

‡*Joint Quantum Institute, University of Maryland and National Institute of Standards and Technology, College Park, Maryland 20742, USA*

¶*Department of Electrical Engineering, Princeton University, Princeton, New Jersey 08544, USA*

E-mail: haa108@pitt.edu

Density Calibration

Here, we compare two independent methods to calibrate the density of the polaritons. The first method uses a photon calibration for our setup that relates the CCD count on the camera to the number of photons detected. This calibration was created by tuning the wavelength of the M Squared laser close to the polariton emission wavelength (810 nm). We then placed a mirror at the sample plane, which reflected the laser to the CCD camera through the same optical set up that was used in the experiment. The total CCD count is related to the number of photons through the equation

$$I_{\mathrm{CCD}} = \frac{1}{\eta}\frac{N_{\mathrm{ph}}}{\Delta t}, \tag{S1}$$

where η is the efficiency factor, Δt is the integration time of the camera and N_{ph} is the number of photons detected by the camera. This can then be related to the number of photons by measuring the power of the laser at the sample plane. The total number of photons the camera detects during an integration time Δt is given by

$$N_{\mathrm{ph}} = \frac{P\Delta t}{hc/\lambda}, \tag{S2}$$

where P is the measured power of the laser at the sample plane, h is Planck constant, c is the speed of light and λ is the wavelength of the laser. The efficiency factor is therefore given by

$$\eta = \frac{P\lambda}{hc}\frac{1}{I_{\mathrm{CCD}}} \tag{S3}$$

This efficiency factor allows us to relate the counts on the camera to the number of photons sent to the camera during a time Δt. The total density of the polaritons for a given I_{CCD} count is then given by:

$$n_{\mathrm{tot}} = \frac{\eta I_{\mathrm{CCD}}\tau}{A_{\mathrm{obs}}}, \tag{S4}$$

where $A_{\rm obs}$ is observation area on the sample, τ is the lifetime of the polaritons. To account for the laser chopping for the PL, we divide by the duty cycle $d = 1.7\%$, which gives

$$n_{\rm tot} = \frac{\eta I_{\rm CCD}\tau}{A_{\rm obs}d}, \tag{S5}$$

The second method that we used to calibrated the density of the polaritons is by fitting the extracted occupation number of the polaritons to the Bose–Einstein distribution. This was done by fitting multiple occupation numbers at different pump powers to the Bose–Einstein distribution using T and μ as fit parameters and a single efficiency factor η. This then constraints the efficiency factor by minimizing the mean-squared error in the fit process. Therfore, for a set of n number of occupation numbers each corresponding to a different pump power, we have a total of $2n + 1$ free parameter, i.e. n temperature parameters, n chemical potential parameters and one single efficiency factor.

The two methods give consistent results. At the threshold of condensation, the photon calibration methods gives a density of $n = 2.97 \pm 1.75$ μm^{-2} while the best fit to the Bose-Einstein distribution gives a density of $n = 3.3 \pm 0.49$ μm^{-2}.

Extracting the occupation

As described in the main text, we used angle-resolved imaging to measure the spectral function $I(k, E)$ of the lower polariton. Figure S1 shows a representative $I(k, E)$ of the lower polaritons at low density. The occupation number is extracted by taking vertical slices at each k value to obtain $I(E)$ for each k slice.This $I(E)$ curve is then fit with a Lorentzian function to extract the polariton energy for each k slice (see for example Fig. S1(b)). The occupation N for each k slice is then related to $I(E)$:

$$N(k_i) = \eta\tau(k_i) \int \mathrm{d}E\, I(k_i, E), \tag{S6}$$

where $\tau(k)$ is the k-dependent radiative lifetime, and η is an overall constant that can be determined by minimizing the mean-squared error in fitting a set of distributions $N(E)$. Since the polaritons are photonic and in the weak coupling regime, the lifetime is not a strong function of k and can be taken as a constant $\tau(k) \sim \tau$.

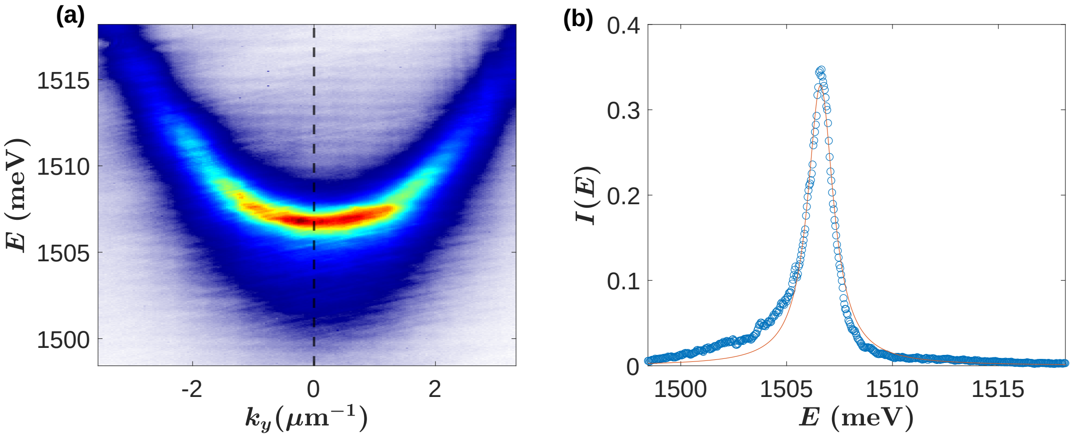

Figure S1: **Method of extracting the occupation.** (a) A typical energy dispersion of the thermalized polaritons. (b) A vertical slice in (a) at $k_y = 0$ showing the CCD counts. The red line is a fit to a Lorentzian function predicting an energy 1.5066 meV for the polaritons at $k_y = 0$. The occupation number for this energy is proportional to the integral of the $I(E)$ curve.

Exciton energy and linewidth

We have measured the exciton PL at room temperature for a sample with bare GaAs quantum wells of the same width as in our microcavity sample, grown on a substrate at Princeton using the same method. Figure S2 shows the PL $I(k, = 0, E)$ using a non-resonant excitation. We see two-peaks– the low energy peak corresponds to the heavy-hole exciton and the high energy peak is the light-hole exciton. We performed a two-Lorentzian fit to extract the energy and linewidth. The heavy-hole exciton has an energy of $E_0 = 1501.3$ meV and a linewidth of 14.6 meV, while the light-hole exciton has an energy of $E_0 = 1525.2$ meV and a linewidth of 49.1 meV. Interestingly, the ratio of the integral of the two Lorentzians is ~ 0.43, which is approximately equal to ratio of heavy-hole excitons to light-hole exciton

given by the thermal Boltzmann factor $\exp(-\Delta E/k_B T) \sim 0.4$, where $\Delta E \approx 23.9$ meV and $T \approx 300$ K. To confirm the energy position of the excitons in the quantum wells inside the

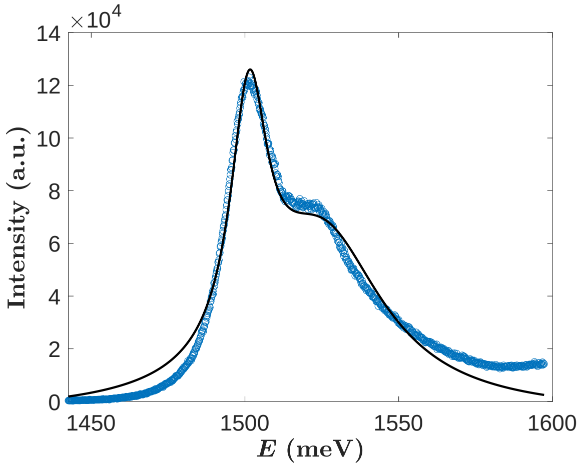

Figure S2: **Exciton energy.** The PL intensity $I(k=0,E)$ of excitons in quantum wells of the same design, but not in a cavity, at room temperature. The solid black is a two-Lorentzian fit for the heavy-hole and light-hole excitons.

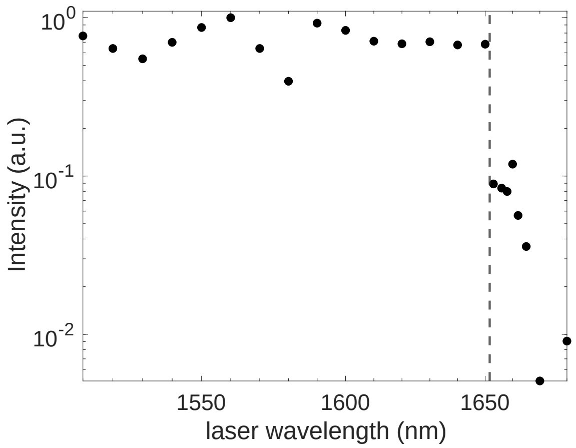

Figure S3: **Two-photon excitation.** Total integrated polariton PL intensity as a function of the pump wavelength. The dashed line corresponds to one half of energy of the heavy-hole exciton shown in Fig. S2.

cavity, we have measured PL of the polariton gas using two-photon excitations for different pump wavelengths. For each pump wavelength, we measured the intensity $I(k,E)$ of the polaritons. We then integrated the whole image to obtain the total intensity, which is plotted in Fig. S3. The dashed line in Fig. S3 corresponds to twice the wavelength of the excitons

measured in Fig. S2. When the pump laser has wavelength longer than twice of the exciton wavelength, the intensity decreases sharply. This confirms that the exciton absorption edge inside the cavity is at the same location as seen in the PL of Fig. S2 from a sample with quantum wells of the same design and no cavity.

Temporal Coherence Measurements

We have also measured the coherence time of the polariton gas. By introducing a time delay between the two interferometer arms, we have measured the visibility function $V = (I_{\max} - I_{\min})/(I_{\max} + I_{\min})$. Figure S4 shows the visibility for each time delay. To extract the coherence time, we have fitted the visibility function with a Gaussian, giving a FWHM of 2.45 ps. The data fluctuations in Fig. S4 are due to the fact that the condensate is not temporally stable which can sometime wash out the fringe visibility due to time integration.

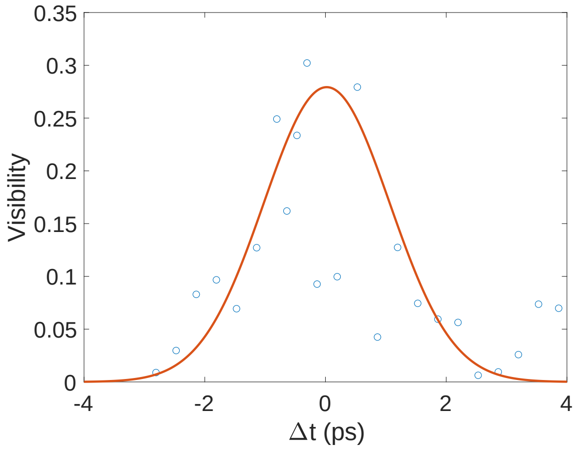

Figure S4: **Coherence time**. A typical visibility curve as a function of the time delay between the two Michelson arms. The red line is a Gaussian fit used to extract the coherence time.

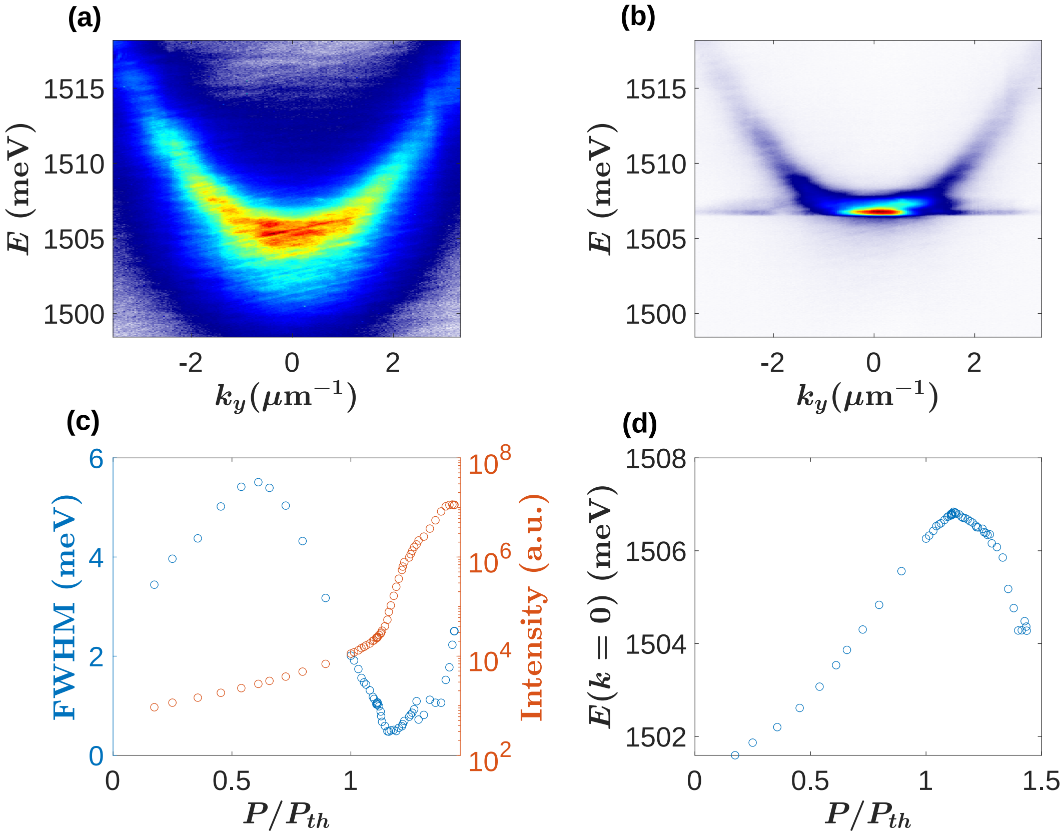

Figure S5: **Blue shift and linewidth narrowing.** (a) the polariton energy dispersion corresponding at $P/P_{th} = 0.73$ and (b) at $P/P_{th} = 1.16$.(c) Full width at half max at $k = 0$ and the intensity as a function of the pump power. (d) the blue shift at $k = 0$ as a function of the pump power.

Polariton linewidth and energy blue-shift

Figure 4 in the main text is from a different location on the sample than the data of Figs 1-3, at which the polaritons have slightly more photonic character. Figure S5 shows the data of the polaritons corresponding to Fig. 4 in the main text. The behavior is quite similar to that of Figs. 1-3 of the main text; in general, we find that the effects reported are easily reproducible over a wide area of the sample. The minimum linewidth we observed for this location on the sample is 0.48 meV. The polaritons remain mostly thermal but we have observed in some cases a secondary condensate mode can appear. This secondary mode is typically much weaker in intensity than the main mode at $k = 0$. Therefore, the polaritons remain in quasi-equilibrium.

Three level model

In this section, we use the three level model discussed in the main paper to simulate $I(E, k)$ for the experimental parameters. A simple three-level model for our GaAs-based microcavity structures which includes heavy-hole excitons, light-hole excitons (see Fig. S2) and photons can be written as

$$H(k) = \begin{pmatrix} E_p + i\Gamma_p & \Omega/2 & \Omega/2 \\ \Omega/2 & E_{\mathrm{hh}} + i\Gamma_{\mathrm{hh}} & 0 \\ \Omega/2 & 0 & E_{\mathrm{lh}} + i\Gamma_{\mathrm{lh}} \end{pmatrix}, \tag{S7}$$

where Γ_p gives half width at half maximum of the photon linewidth broadening, here about $\hbar/(100\ \mathrm{ps}) \sim .01$ meV, and Γ_{hh} and Γ_{lh} give the heavy-hole and light-hole exciton broadening, here set to 7.3 meV and 24.6 meV, respectively. The exciton energies are $E_{\mathrm{hh}} = 1501.3$ meV and $E_{\mathrm{hh}} = 1525.2$ meV. The energies and linewidths of the light and heavy excitons are extracted from Fig. S2. The cavity photon energy is given by

$$E_p = E_{p0} + \frac{\hbar^2 k^2}{2m_p}, \tag{S8}$$

where cavity zero energy is taken to be $E_{p0} = 1.4981$ meV and the cavity mass is set to $m_p = 4.7 \times 10^{-5}\ m_e$, where m_e is the mass of the electron in vacuum.

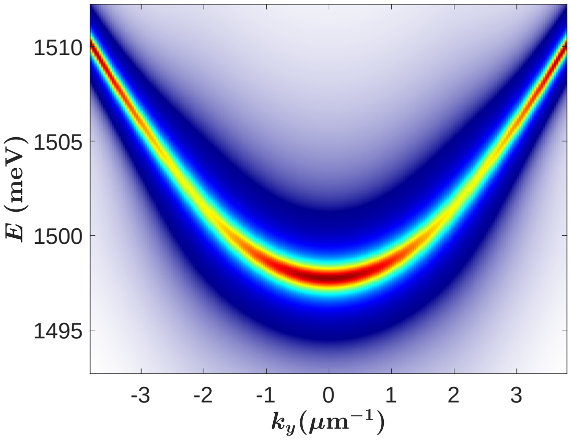

Figure S6: **Simulated energy dispersion.** The energy dispersion from the three level model using $\Omega = 4.5$ meV

We diaonalized the Hamiltonian for each k value to find the real and imaginary energies and the linewidths of the lower and middle polaritons. This then allowed us to compute a simulated $I(k, E)$ image by adding two Lorentzians together with the fit center energies and widths from the eigenenergies at each k. Therefore, the intensity of the lower polariton is given by

$$I_{LP}(E) = \frac{A}{\pi} \frac{\frac{1}{2}\Gamma_{LP}}{(E - E_{LP})^2 + \left(\frac{1}{2}\Gamma_{LP}\right)^2}, \tag{S9}$$

where Γ_{LP} is the full width at half max of the lower polariton and E_{LP} is the lower polariton energy. To insure that the polaritons are in an equilibrium distribution, we enforce that

$$A \int \mathrm{d}E\ I_{LP}(E) = e^{-(E_{LP}-E_0)/k_B T}, \tag{S10}$$

which implies that

$$A = \frac{e^{-(E_{LP}-E_0)/k_B T}}{\int \mathrm{d}E\ I_{LP}(E)}. \tag{S11}$$

To account for the radiative lifetime of the polaritons, we multiply by the photon fraction,

which then gives $N_{LP}(E) = AC_{LP}(k)I_{LP}(E)$. Similarly, for the middle polariton, we have

$$I_{MP}(E) = \frac{A}{\pi}\frac{\frac{1}{2}\Gamma_{MP}}{(E-E_{MP})^2+\left(\frac{1}{2}\Gamma_{MP}\right)^2}. \tag{S12}$$

Accounting for the thermalization factor and the radiative lifetime, $N_{MP}(E) = BC_{MP}(k)I_{LP}(E)$, where B is given by

$$B = \frac{e^{-(E_{MP}-E_0)/k_BT}}{\int \mathrm{d}E\ I_{MP}(E)}. \tag{S13}$$

The total intensity is then given by

$$I_{\text{tot}} = N_{LP}(E) + N_{MP}(E). \tag{S14}$$

Figure S6 shows the simulated $N(k, E)$ image. For this fit, the total exciton fraction (light hole plus heavy hole) is 8.5% at $k = 0$. Clearly, the polaritons are in the weak coupling regime since the linewidth of the exciton is larger than the Rabi splitting. However, since the exciton resonance lies near to the energy of the photon mode, strong nonlinearity can be observed as reported in the main text.

Estimation of Q factor

The cavity lifetime of our samples has been measured at low temperature, giving a cavity lifetime of $\sim$ 135 ps.[1] In this section, we show by using the transfer-matrix calculations that the cavity lifetime is not a strong function of the temperature. We have performed transfer-matrix calculations of the Q factor of the cavity using room-temperature values of the indices of refraction of the materials (see Fig. S7), and Q increases from 2.3105×10^5 at 10 K to 2.6105×10^5 at 300 K. The details of transfer-matrix calculations is given in reference.[2]

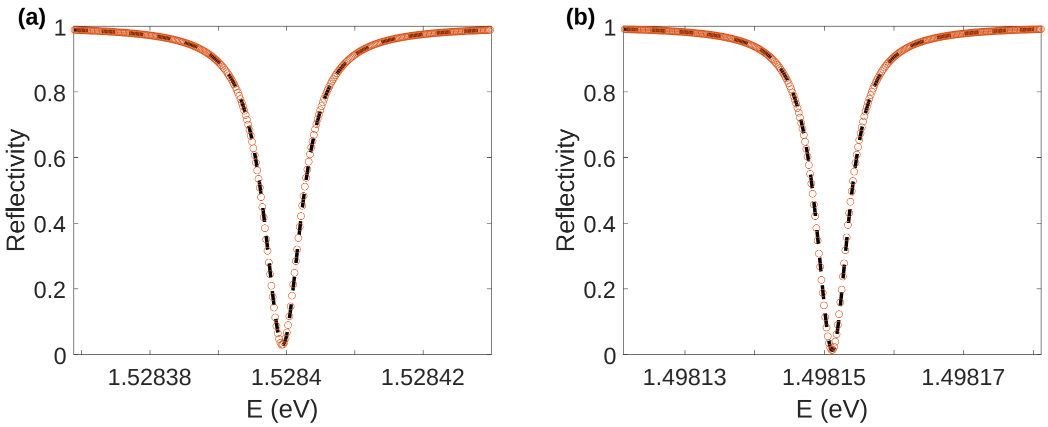

Figure S7: **Simulated reflectivity of the cavity mode.** (a) circles: the reflectivity of the cavity mode at $T = 10k$. Dashed line: Lorentzian. (b) circles: the reflectivity of the cavity mode at $T = 300k$. Dashed line: Lorentzian fit. The Q factor is extracted from the Lorentzian fits using the peak energy E_{cav} and the FWHM to calculate $Q = E_{\text{cav}}/\text{FWHM}$.

References

(1) Steger, M.; Gautham, C.; Snoke, D. W.; Pfeiffer, L.; West, K. Slow Reflection and Two-photon Generation of Microcavity Exciton–polaritons. *Optica* **2015**, *2*, 1–5.

(2) Beaumariage, J.; Sun, Z.; Alnatah, H.; Yao, Q.; Myers, D. M.; Steger, M.; West, K.; Baldwin, K.; Pfeiffer, L. N.; Tam, M. C. A.; others Measurement of exciton fraction of microcavity exciton-polaritons using transfer-matrix modeling. *2024, arXiv:2406.12940. ArXiv preprint.* `https://arxiv.org/abs/2406.12940` (accessed 12/17/2024).
